# Rapid absorption of frontal lobe contusion and laceration with hematoma: A case report and review of literature

**DOI:** 10.1097/MD.0000000000033522

**Published:** 2023-05-05

**Authors:** Yangzong Wu, Penghui Liu, Yuanxiang Lin, Shuiqing Qian, Xiaoxin Chen, Zhiquan Fan

**Affiliations:** a Department of Neurosurgery, The Second Hospital of Longyan, Fujian Province, Longyan, China; b Department of Neurosurgery, The First Affiliated Hospital of Fujian Medical University, Fuzhou, China.

**Keywords:** case report, cerebral hematoma, rapid absorption

## Abstract

It is rare for a traumatic intracranial hematoma to self-absorb rapidly after conservative treatment. To the best of our knowledge, there has been no report in the relevant literature of rapid absorption of hematoma formation following cerebral contusion and laceration.

**Patient concerns:**

A 54-year-old male was admitted to our hospital with head trauma at 3 hours prior to admission. He was alert and oriented, glasgow coma scale score of 15. Head computed tomography (CT) showed left frontal brain contusion with hematoma, however, a reexamination of CT about 29 hours following the trauma revealed that the hematoma had been absorbed.

**Diagnoses:**

A diagnosis of contusion and laceration of left frontal lobe with hematoma formation was made based on the CT images.

**Interventions:**

The patient underwent conservative treatment.

**Outcomes:**

After treatment, dizziness and headache subsided for the patient, and no special discomfort was reported.

**Lessons:**

It is likely that the reason for rapid absorption in this case is that the hematoma is prone to liquefaction because of abnormal platelet values and coagulation dysfunction. As the liquefaction hematoma breaks into the lateral ventricle, it is redistributed and absorbed in the lateral ventricle and subarachnoid space. Further evidence is required to support this hypothesis.

## 1. Introduction

It is more common for frontal lobe hematomas to be caused by frontal lobe contusions or lacerations during craniocerebral trauma, most often in combination with injuries to the occipital region. Having an uneven anterior skull base, falx cerebrum, sphenoid crest and coronal structure protruding from intracranial. It is easy to cause offset injuries to the bottom and medial frontal lobes following deceleration injury, and bilateral frontal lobe brain contusion injuries are more common. As a result of the contusion and laceration of the frontal lobe, timing of the surgery is difficult to determine, and often the condition progresses rapidly, leading to the development of a central cerebral hernia. For conservative treatment of frontal lobe hematoma, it often takes several weeks for the hematoma to be completely absorbed, which can lead to neuropsychiatric symptoms. Here we present a case of frontal lobe contusion and laceration with hematoma with rapid absorption, which was treated in our hospital. Following are the details of the case and a literature review was conducted in order to explore the possible explanations for rapid hematoma absorption.

## 2. Case report

A 54-year-old male suffered a fall and landed on his occipital area at 3 hours prior to admission, causing pain and bleeding in his occipital area. This patient had been diagnosed with “epilepsy” in other hospitals for more than 10 years, and had taken sodium valproate sustained release tablet 250 mg twice a day for a long time with satisfactory results.

**Figure 1.**
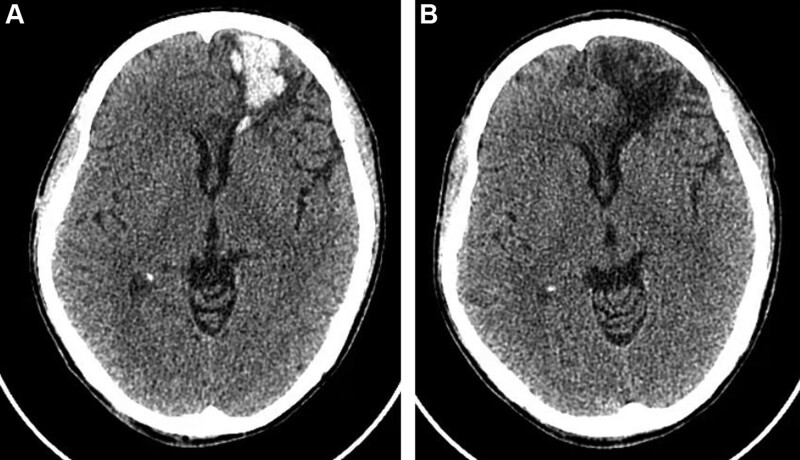
Head CT findings of left frontal brain contusion with hematoma, (A) head CT findings about 3 hours after trauma, (B) CT findings of the head at about 29 hours after trauma. CT = computed tomography.

Physical examination: alert and oriented to person, place, time, and situation, soft neck, no abnormality in heart, lung, and abdomen, glasgow coma scale score of 15 (E4V5M6), bilateral pupils are at the same size, 3 mm in diameter, sensitive to light reflection. Normal muscle strength and tension of limbs. Negative Babinski. It was noted that the occipital scalp had a 1.0 cm long laceration with foreign body contamination and some bleeding. Auxiliary examination on admission showed: blood routine: White blood cell 9.25 × 10^9^/L, red blood cell 4.85 × 10^12^/L, hemoglobin concentration 139 g/L, platelet 101 × 10^9^/L↓. Coagulation test: prothrombin time 10.5 seconds, national standard ratio 0.91, activated partial thrombin time 21.6 seconds, thrombin time 20.0 seconds, fibrinogen 1.63 g/L↓. Head computed tomography (CT) examination: contusion and laceration of left frontal lobe with hematoma formation (Fig. 1A). Diagnosis: Contusion and laceration of left frontal lobe with hematoma formation; Contusion and laceration of occipital scalp. A variety of treatments were administered to the patient following admission, including wound debridement and suturing, infection prevention, fluid replacement, hemostasis, epilepsy prevention, and other symptomatic treatments. A day after admission, the patient did not report any special discomfort, and dizziness and headache subsided after treatment. A reexamination of the head CT about 29 hours following the trauma revealed that the hematoma had been absorbed (Fig. 1B). The written informed consent was obtained from the patient.

## 3. Discussion

It is rare for a traumatic intracranial hematoma to self-absorb rapidly after conservative treatment, and self-absorption usually takes weeks or even months. According to current research, most scholars define the rapid absorption of traumatic intracranial hematoma as the majority absorption or complete absorption of intracranial hematoma within 72 hours after trauma.^[1]^ In our view, a rapid absorption of hematoma requires 3 conditions: the hematoma must be rapidly liquefied, and the liquefied hematoma has a smooth outflow channel and a clear direction.

There are several factors that may contribute to rapid absorption. In patients with abnormal coagulation function, traumatic intracranial hematomas tend to increase, resulting in aggravation of the condition. However, it has been reported that traumatic intracranial hematomas are rapidly absorbed in patients with abnormal coagulation may be due to the fact that the abnormal coagulation hematoma are easy to dilute and liquefy, and the liquefied hematoma are easily expelled. Thus, this satisfies the first condition for rapid absorption of the hematoma in our case, ^[2,3]^ which presented with low fibrinogen and platelets upon admission to the hospital. There is a possibility that abnormal coagulation may be associated with long-term oral valproate use or traumatic coagulopathy. Other scholars believe that thrombolysis enzymes are more active in patients who have experienced rapid absorption of traumatic intracranial hematoma. However, it has not been determined whether thrombolysis enzymes are activated in this patient.^[4]^

Skull fracture has been reported to affect the rapid absorption of traumatic intracranial hematoma, which often occurs in patients after craniocerebral trauma. In some patients, the dura and arachnoid membrane may be torn after skull fracture, resulting in diluted intracerebral hematoma or subdural hematoma that can flow into the epidural and be absorbed subcutaneously.^[3]^ When a traumatic subdural hematoma occurs with senile brain atrophy, more cerebrospinal fluid (CSF) flows into the subdural space after an arachnoid laceration due to the widening of the subdural space, which allows the hematoma to be diluted and absorbed more easily.^[5]^ The patient in this case was a middle-aged male who suffered contusions and lacerations of the frontal lobe caused by typical hedging injuries and did not exhibit any obvious signs of brain atrophy or fracture of the skull.

Hematoma absorption is enhanced by arachnoid laceration, diluting CSF, flushing the hematoma and redistributing the hematoma. The CT examination of patients with rapid absorption of traumatic intracranial hematoma revealed numerous low-density areas between the internal plate of the skull and the hematoma and/or extensive subarachnoid hemorrhage, which may be a manifestation of CSF scouring the hematoma. ^[3,5–7]^ The hematoma in this patient was homogeneous, but penetrated into the lateral ventricle frontal horn (arrow in Fig. 1A). It may be that the liquefied hematoma enters the CSF circulation through the lateral ventricle frontal horn as a mechanism of absorption.

It is common for cerebral edema to occur following a craniocerebral injury, and the edema brain tissue presses the hematoma, causing the hematoma to redistribute into the subdural and subarachnoid spaces. When the hematoma mixes with CSF, it is diluted in the subarachnoid space and absorbed.^[8]^ Otherwise, the hematoma may be forced into the subgaleal space through the torn dura and fracture suture and absorbed from there. It has even been suggested that the repeated fluctuations in intracranial pressure caused by frequent irritability or nausea and vomiting may contribute to the rapid absorption of the hematoma.^[7]^ However, this patient did not exhibit any obvious signs of cranial hypertension or cerebral edema on imaging following admission; thus, cerebral edema is not the cause of rapid absorption in this case.

Although there is a significant amount of bleeding and even midline displacement in some patients with rapid absorption of traumatic intracranial hematomas, the clinical symptoms are usually mild, and there are usually no obvious signs of neurological improvement on the neurological examination.^[5]^ There was an obvious cerebral contusion and laceration with hematoma formation in this patient, but he was not experiencing any dizziness or headache at the time of admission, and no signs of dizziness or headache were noted after 1 day of treatment. However, there are only a few patients with rapid absorption of hematoma clinically, and it is difficult to predict which patients are prone to rapid absorption of hematoma, even if patients with obvious characteristics of rapid absorption of hematoma still need to closely observe the changes. As part of conservative treatment, regular imaging examinations are required to assess the condition.^[9]^ Conservative observation and treatment should be cautious for patients with no obvious clinical symptoms but with imaging examination of patients with surgical evidence.

There has been no report in the relevant literature of rapid absorption of hematoma formation following cerebral contusion and laceration. It is likely that the reason for rapid absorption in this case is that the hematoma is prone to liquefaction because of abnormal platelet values and coagulation dysfunction. As the liquefaction hematoma breaks into the lateral ventricle, it is redistributed and absorbed in the lateral ventricle and subarachnoid space. Whether the thrombolysis enzyme system is abnormal in the body requires further confirmation.

## Author contributions

**Conceptualization:** Shuiqing Qian, Xiaoxin Chen, Zhiquan Fan.

**Investigation:** Yangzong Wu, Penghui Liu.

**Methodology:** Penghui Liu, Zhiquan Fan.

**Project administration:** Zhiquan Fan.

**Resources:** Yangzong Wu.

**Supervision:** Shuiqing Qian, Xiaoxin Chen, Yuanxiang Lin.

**Writing – original draft:** Yangzong Wu, Penghui Liu.

**Writing – review & editing:** Yuanxiang Lin.
